# Shedding New Light on Ancient Glass Beads by Synchrotron, SEM-EDS, and Raman Spectroscopy Techniques

**DOI:** 10.1038/s41598-019-52322-2

**Published:** 2019-11-05

**Authors:** Seriwat Saminpanya, Chatree Saiyasombat, Nirawat Thammajak, Chanakarn Samrong, Sirilak Footrakul, Nichanan Potisuppaiboon, Ekkasit Sirisurawong, Thumrongsak Witchanantakul, Catleya Rojviriya

**Affiliations:** 1 Department of General Science, Faculty of Science, Srinakharinwirot University, 114 Sukhumvit 23 Road, Khlong Toey Nua, Watthana, Bangkok 10110 Thailand; 2 Synchrotron Light Research Institute, 111 University Avenue, Suranaree Sub-District, Muang District, Nakhon Ratchasima, 30000 Thailand; 3 School of Chemistry, Institute of Science, Suranaree University of Technology, 111 University Avenue, Suranaree Sub-District, Muang District, Nakhon Ratchasima, 30000 Thailand

**Keywords:** Geochemistry, Mineralogy

## Abstract

The oxidation states of colouring elements and the pigments in ancient rare glasses have been investigated in this study. Synchrotron X-ray, SEM-EDS, and Raman techniques revealed that Cu^2+^plays a major role in blue and green glasses. The lead stannate pigment gives glasses a yellow colour. Copper and lead stannate can cause the green colour in glasses, and iron gives rise to the colour of black glasses. Microcomputed tomography reveals the distribution of the heavy elements, pigments, and inclusions in the glasses. The Dvaravati glasses in Southeast Asia may have been imported or technologically transferred to domestic manufacturers during trading on the Silk Road that connected the East and the West.

## Introduction

Glass can be any colour and is usually made by melting quartz sand (SiO_2_) at a temperature of approximately 1000 °C together with calcium oxide (CaO), soda (Na_2_O), and/or potash (K_2_O). The alkalis – soda and potash – play the role of lowering the melting point of the silica while calcium oxide stabilizes the mixture. Soda glass was made in the Roman period, while potash glass was more common in the Middle Ages^1^. Several metal salts were used as colourants, e.g., Co creates blue colour, while Cu creates green colour. Fe-oxides are usually present as impurities in the sand and can often create a greenish or bluish coloured glass. Mn and/or Sb were added to the melt to make a colourless glass^1^. Pb can make the glass more shiny and translucent^2^. Other elements, e.g., Al, Zr, and Sn can also be found in ancient glasses. In the past, glass makers would deliberately add some compounds to melt the ingredients. However, some other elements could be added unintentionally because they were present in the form of an inclusion in the compound, or perhaps, the hidden purposes of these elements have been lost with time. This is especially true regarding the information about the colouring elements and compounds, and the oxidation states of the transition colouring element.

Investigation of the glasses examined in this work proved that different colours contain a high level of additional elements, e.g., yellow for Pb, blue for Cu, green for mixed Cu and Pb, and black for Fe^3^. However, the species and types of compounds formed in the matrix glass, their structure, and the theoretical explanation of the origins of the colours in ancient glasses are still an open problem. For example, Pb may be a component of yellow pigment compounds such as orthorhombic lead-tin yellow I, lead (II) stannate (Pb_2_SnO_4_), cubic lead-tin yellow II (PbSn_1−x_Si_x_O_3_) or cubic lead-tin antimonite (Pb_2_Sb_2x_Sn_x_O_6.5_)^4^. Cu^2+^ is believed to be the origin of blue and greenish-blue colours as indicated by the UV–Vis–NIR band excitations that were identified as due to a d-d transition^5^ in the octahedral coordination of crystal field theory. A few works, e.g., the studies by Klysubun *et al*.^6^ and Ravel *et al*.^7^ studied more recent examples of historical glasses. Li *et al*.^8^ used SEM-EDS and Raman microscopy to investigate the crystalline pigment in ancient glass beads. Laser abrasion-inductively coupled plasma-mass spectrometry (LA-ICP-MS) was used to analyse the chemical composition of these samples in previous work^3^, but the average values from several spots were still not the best representative of the bulk composition for this inhomogeneous sample fabricated by an ancient technique. The older examples, such as our samples, needed further investigations of the oxidation states and information regarding the pigments of these glasses that are not reported in the literature. The present work provided knowledge of the compound, refining the information regarding the origins of the colour of ancient glass beads and the mechanisms of the compound interacting with matrix materials. The present work is a multidisciplinary study of geochemistry, archaeological science, mineralogy, and spectroscopy. Multiple techniques were used to study the evidence of pigments and inclusions that were revealed by scanning electron microscopy (backscattered electron image and elemental mapping) to observe the elemental distribution, and computed tomography (micro-CT) was used to observe the pigments and inclusion distribution inside the sample, while chemical composition analyses of bulk using X-ray fluorescence (XRF), and Raman spectroscopy was used to determine the nature of bonding. The technique used for glass production in ancient times was revealed. The chemical compositions in terms of cation speciation for some colouring elements in the glasses of highland Pang Mapha, Mae Hong Son province and in the lowland Sa Kaeo province of Thailand have also been reported. This could help to provide information about the provenance of the Dvaravati glasses in this region that may be imported or technologically transferred to a domestic manufacturer.

## Results and Discussion

### XRF

**Figure 1:**
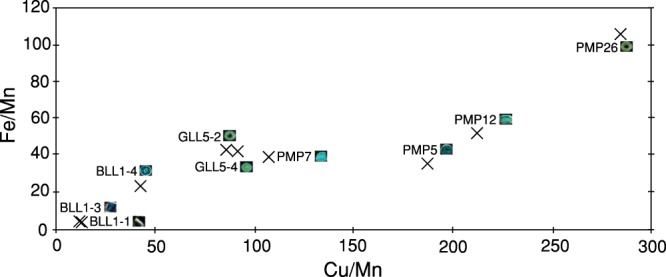
XRF results from beamline BL1.1W for ancient glass beads shown by plotting the Fe/Mn ratios against the Cu/Mn ratio, which can discriminate between the beads in terms of different colours and localities.

The semi-quantitative XRF results for the Fe/Mn ratio were plotted against the obtained Cu/Mn ratio values, showing that the yellowish green sample had higher Fe and Cu contents. Deep blue samples, i.e., BLL-1, BLL1-3, and BLL1-4 were found to be depleted of these elements (Fig. 1). The Pang Mapha beads (PMP-5, PMP-7, PMP-12, and PMP-26) clearly showed Cu-rich contents compared to those from Sa Kaeo. Therefore, the following conclusions can be drawn:

1. Cu contents were 100 times greater than Mn contents, implying that the Cu/Mn ratio of 100 can differentiate between the localities of the glasses in the study.
2. The beads from La Lu with the blue colours had Cu/Mn and Fe/Mn ratios in the range of 11.9–42.1 and 3.6–23.1, respectively, while the green samples had Cu/Mn and Fe/Mn ratios that increased to 85.6–91.7 and 41.9–42.9, respectively.
3. The Pang Mapha glass beads that varied from blue to green had the Cu/Mn and Fe/Mn ratios in the ranges of 106.9–187.1 and 35.4–39.3, respectively, or the Fe/Mn ratio increased in the greener samples that raised the Cu/Mn and Fe/Mn ratios from 211.5–283.8 to 51.7–106.2, respectively.
4. The blue colour of the samples can be gradually changed to the green colour by increasing the Cu/Mn and Fe/Mn ratios.

The area under the curve of Cu and Fe fluorescent peaks provided interesting Cu/Fe ratio values for some of the samples. The BLALL5-1, BLALL5-3, and BLALL5-4 samples were depleted in Cu but enriched in Fe. Sample YLL5-4 was analysed by the X-ray beam hitting 2 spots that were black and yellow and exhibited the Cu/Fe ratios of 0.53 and 2.791, respectively. Their values were too low to be shown in the graph. Samples BLL-1, BLL1-3, BLL1-4, and PMP12 were enriched in Cu. Thus, it can be concluded that Cu gives rise to the blue and green colours in these samples.

### XANES

#### Cu

**Figure 2:**
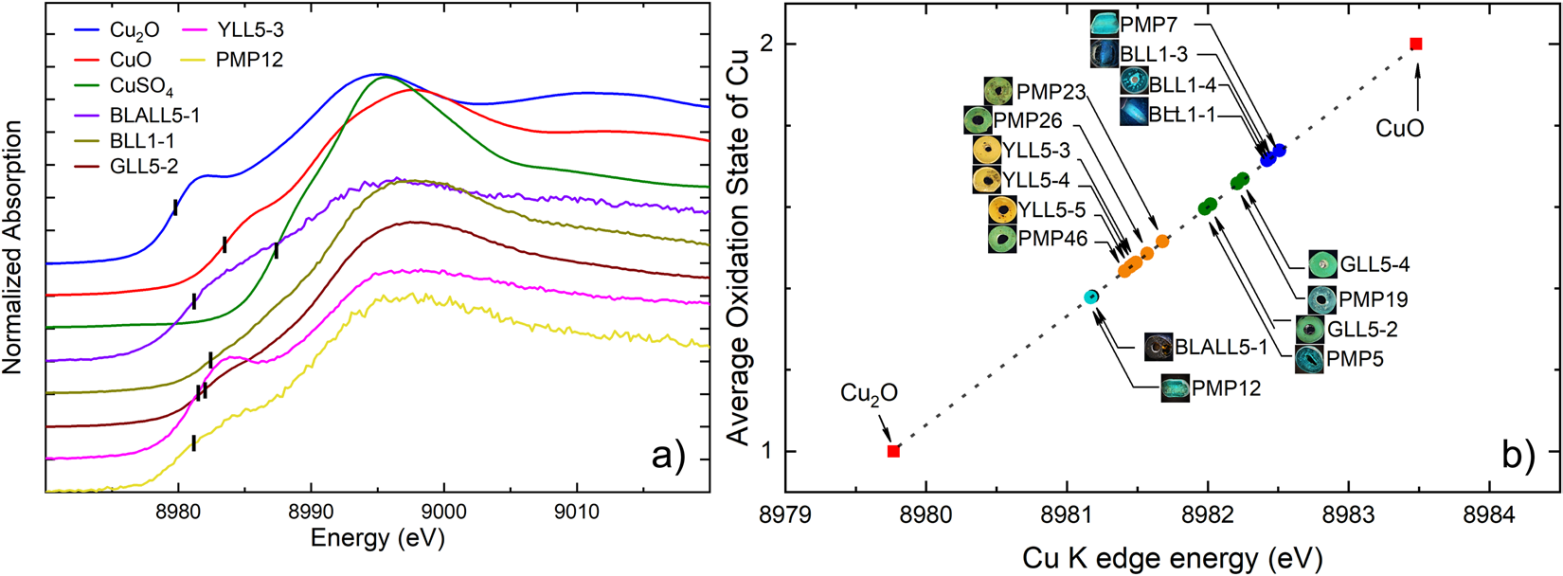
Glass samples divided into five groups according to their average Cu oxidation state: (**a**) Group I: BLALL5-1, black, shows the peak matching the Cu_2_O standard and indicates the Cu^+^ content; Group II: BLL1-1, BLL1-3, BLL1-4, and PMP-7, deep blue, show a best fit to the CuO standard or Cu^2+^; Group III: GLL5-2, GLL5-4, PMP-5, and PMP-19, green and greenish blue, correspond to CuO, i.e., Cu^2+^ as in group II; Group IV: YLL5-3, YLL5-4, and YLL5-5, yellow, PMP23, PMP26, and PMP46, yellowish-green, had a higher pre-edge and tended to be located closer to the Cu_2_O or Cu^+^; and Group V: PMP12, greenish-blue, showed copper in the form between Cu^+^ and Cu^2+^ (all peaks are shown in Supplementary Information); (**b**) Summary of the average oxidation states and the results for the *K*-edge energy of Cu derived from the experiments (see Tables in the Supplementary Information for the absolute oxidation state values), corresponding to the marks in (**a**). The line shows an interpolation between these two reference compounds.

The observed oxidation states of Cu found in the samples ranged between 1 and 2 (Fig. 2). The absorption edge position were evaluated from the first derivative of XANES spectra and marked in Fig. 2a and corresponded to a 1 *s* → 4*p* transition. The accuracy of absorption edge determination from the first derivative of XANES spectra was about 0.05 eV. The reference absorption edges of the +1 and +2 oxidation states were plotted according to Cu_2_O and CuO standards, respectively (Fig. 2b) and the oxidation states of each sample were interpolated between these two references to show the range of oxidation state. The oxidation state values reported for the samples were not integers because the Cu-atoms are affected by the surrounding atoms of other elements resulting in the mixture of oxidation states. The distorted octahedral environment of Cu with longer Cu-O bonds along the z-direction led to the common features of the Cu *K*-edge XANES spectra. Peak *A1* corresponds to the 1 *s* → 4*p*_*xy*_ transition while peak *B1* is related to the 1 s → 4*p*_*z*_ transition^9,10^. Based on the absorption edge positions and features of the spectra, the samples were classified into 5 groups as follows:

1. Group I: For black colour sample BLALL5-1, the absorption edge position was closer to that of the Cu_2_O standard and indicated that the average oxidation of the Cu species was close to +1.
2. Group II: Deep blue samples, BLL1-1, BLL1-3, and BLL1-4, and PMP-7. These samples have similar colour, even though they are obtained from different localities. The absorption edge of this group was between those of Cu_2_O and CuO. Therefore, the samples in this group contain mixed proportions of monovalent and divalent copper.
3. Group III: Green and blue samples, GLL5-2, GLL5-4, PMP-5, and PMP-19. Similar to group II, this group corresponded to the mixture of Cu^+^ and Cu^2+^ but the *A1* peak had a higher intensity and the average oxidation state was closer to Cu^+^ than for the samples in Group II.
4. Group IV: The yellow samples, YLL5-3, YLL5-4, and YLL5-5 and the yellowish-green samples, PMP23, PMP26, and PMP46 had the most intense *A1* peak and the average oxidation state contained more Cu^+^ than the samples in groups II and III.
5. Group V: The PMP12 sample was greenish-blue and was a mixture of blue from the samples in group II and green from the samples in group III, but its absorption edge position was similar to those of the samples in group I because Cu^+^ was dominant.

Generally, Cu is added as a blue colouring element^11^ and the blue-green colour materials made in ancient furnaces were found to be in a reduced condition^12^. In this study, a similarity was found between the Cu colourant and the glasses from other localities such as late 17^th^-century to early 20^th^-century beads from Ontario, Canada, and the eastern Great Lakes region^13,14^. The Cu^2+^ in the Egyptian glasses was studied, and it was suggested that chalcanthite (CuSO_4_·5H_2_O) was most likely deliberately added to the melt provided a blue colour^15^. This is in agreement with the results of this study that copper II was found in the blue and green samples.

#### Fe

**Figure 3:**
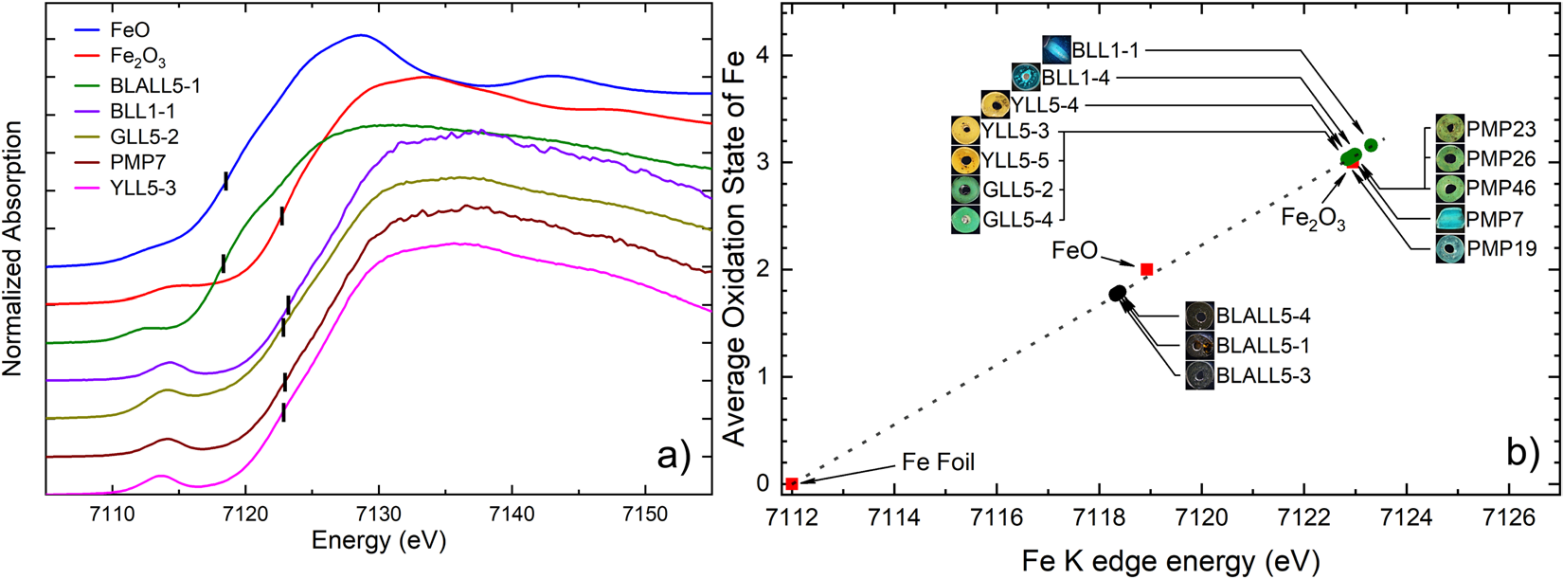
Glass samples divided into two groups according to average Fe oxidation state: (**a**) Group I: BLALL5-1, BLALL5-3, and BLALL5-4, black, show that Fe is found in the form of FeO or Fe^2+^; and Group II: samples with other colours (yellow, green, and blue) demonstrate that their peaks were all matched with the Fe_2_O_3_ or Fe^3+^ peak (all peaks are shown in Supplementary Information); (**b**) The plot shows a summary of the average values for the Fe oxidation states plotted against the *K*-edge energy derived from the experiments, corresponding to the marks in (**a**). The values were not in integer form, showing that the Fe-atom was affected by the surrounding atoms of other elements. The line shows a linear fit with the reference compounds.

The pre-edge feature, corresponding to the 1 *s* → 3*d* transition, and the absorption edge position, corresponding to the 1 *s* → 4*p* transition, were observed and marked in Fig. 3a. The reference absorption edges of the 0, + 2, and + 3 oxidation states were plotted according to the Fe metal foil, FeO and Fe_2_O_3_ standards, respectively (Fig. 2b). The calibration line (dot plot in Fig. 2b) was created from linear fit of these three reference standards. The Fe in the samples can be classified into 2 groups as follows (Fig. 3).

1) Group I: All of the black samples, namely, BLALL5-1, BLALL5-3, and BLALL5-4 were compared with goethite, FeO, and Fe_2_O_3_. The results showed that Fe in the samples is present in the form of Fe^2+^. It can be fundamentally concluded that Fe^2+^ causes the black colour in the samples. This suggests that the glass makers may have added pyrite (FeS_2_) as the ingredient because Fe-S is a chromophore^16^.

2) Group II: For the samples with other colours (yellow, green, and blue), it was found that all of their peaks were matched to the peak of goethite (Fe^3+^). It can also be suggested that Fe^3+^ does not principally cause their colours in this group.

#### Mn

**Figure 4:**
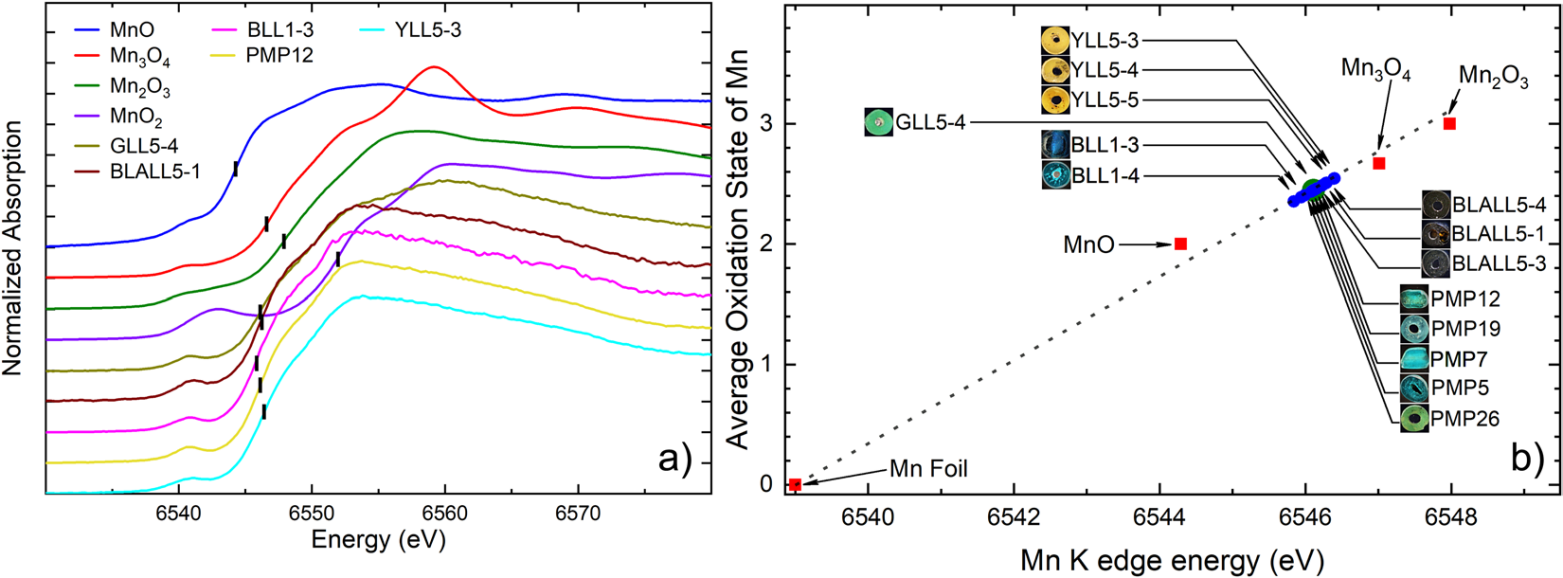
Glass samples divided into two groups according to average Mn oxidation state: (**a**) Group I: GLL5-4, green, exhibits manganese in the form between Mn^2+^ and Mn^3+^; and Group II: samples other than those in group I had the same energy pattern as XANES and showed the pattern peaks that were between MnO and Mn_3_O_4_ (all peaks are shown in Supplementary Information); (**b**) A summary of the average Mn oxidation states and the *K*-edge energy obtained from the experiments, corresponding to the marks in (**a**). The line is a linear fit from the reference compounds.

All of the samples showed similar peak patterns and positions and 2 peaks (*A2* and *B2*) were observed for each sample. The average oxidation states of manganese found in the samples ranged from 2.4 to 2.6 (Fig. 4), slightly below the average oxidation state of Mn_3_O_4_. Therefore, the Mn in the beads was due to the mixture of Mn^2+^ and Mn^3+^. The samples are divided into 2 groups, as follows:

1. Group I: A green sample, GLL5-4, contained manganese with the oxidation state between Mn^2+^ and Mn^3+^. The second peak (*B2*) at approximately 6560 eV had a higher absorption intensity than the first peak (*A2*). This green sample from La Lu, GLL5-4 had different surrounding neighbors compared to other green samples such as one from Pang Mahpha, PMP-12. Manganese must be surrounded by other elemental atoms in a silica environment. Furthermore, this group does not show a pattern similar to that of the group II samples.
2. Group II: Other samples showed similar features in their XANES spectra. In fact, their absorption edges were located between those of MnO and Mn_3_O_4_. In this group, peak *A2* had a higher intensity than peak *B2*.

Generally, Mn was used as a decolourant for ancient glasses^17^ and pyrolusite (MnO_2_) may have been added and this process was well-characterized in Roman glasses^18,19^. However, the MnO: Fe_2_O_3_ ratio was very low^3^, indicating that Mn was not intentionally added as a decolourant^17^. This also proves that Mn was not a major colourant.

### SEM-EDS

SEM-EDS measurements provided backscattered electron images that revealed the texture and inclusions of the glasses. The spectra of the qualitative chemical composition can be readily obtained by several spot analyses in a very short time. The instrument also provided elemental mapping results for the flat surface of the samples.

**Figure 5:**
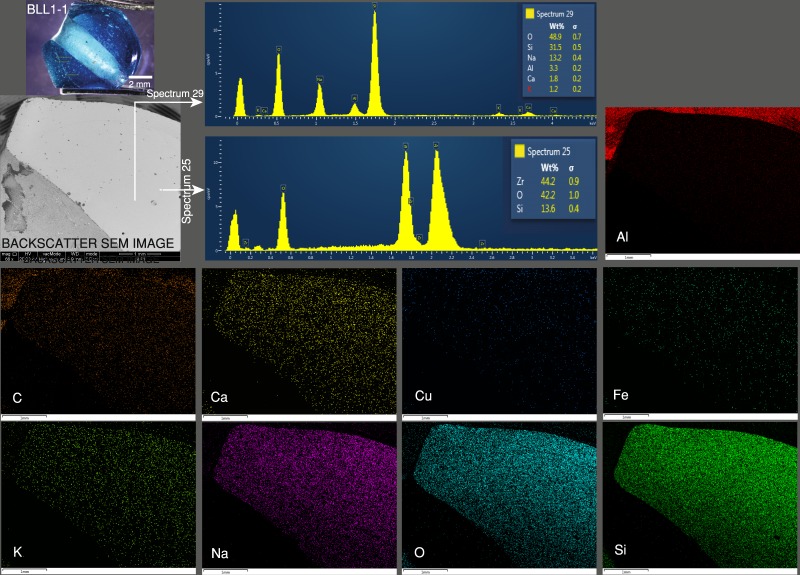
The blue sample, BLL1-1, has a smooth and homogeneous texture with few inclusions. Spectrum 29 shows the major element composition. Spectrum 25 reveals a zircon (ZrSiO_4_) inclusion. Elemental mapping indicated the major and some trace elements.

The blue sample, BLL1-1, (Fig. 5) has a smooth and homogeneous texture with few inclusions. Spectrum 29 in the figure was obtained from the beam hitting a clean surface and showed the major element composition of soda-alumina glass, including the O, Si, Na, Al, and Ca material. Spectrum 25 was obtained from a bright scattering inclusion and revealed a zircon (ZrSiO_4_) inclusion. Elemental mapping indicated major and some trace elements, and in particular the Cu colouring element was distributed throughout the glass matrix in a very small amount.

**Figure 6:**
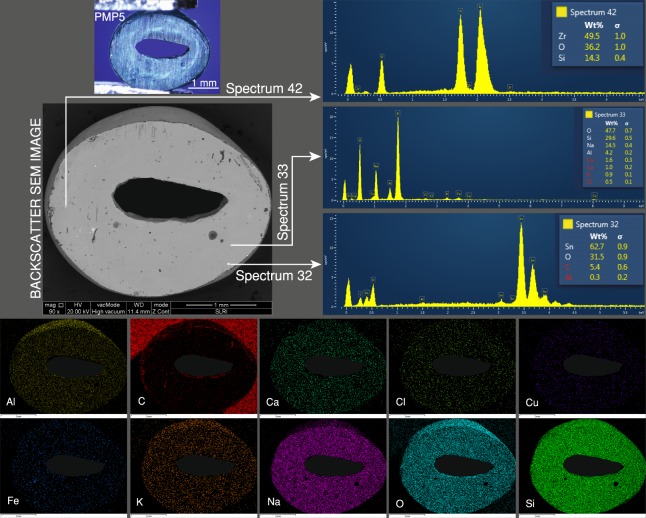
The BSE image of the greenish-blue PMP5 sample, showing some bright scattered tiny inclusions. Spectra 32 and 42 indicate cassiterite (SnO_2_) and zircon (ZrSiO_4_) inclusions, respectively. The glass matrix is represented by spectrum 33. The Cu is evenly distributed throughout the glass matrix.

The greenish-blue PMP5 (Fig. 6) shows a relatively clean polished surface as observed from the BSE image with slightly bright scattered tiny inclusions. Spectra 32 and 42 in the figure were obtained from two different bright scattered tiny inclusions representing cassiterite (SnO_2_) and zircon (ZrSiO_4_), respectively. The soda-alumina glass matrix is represented by spectrum 33, and includes O, Si, Na, and Al. Cu was evenly distributed throughout the glass matrix.

**Figure 7:**
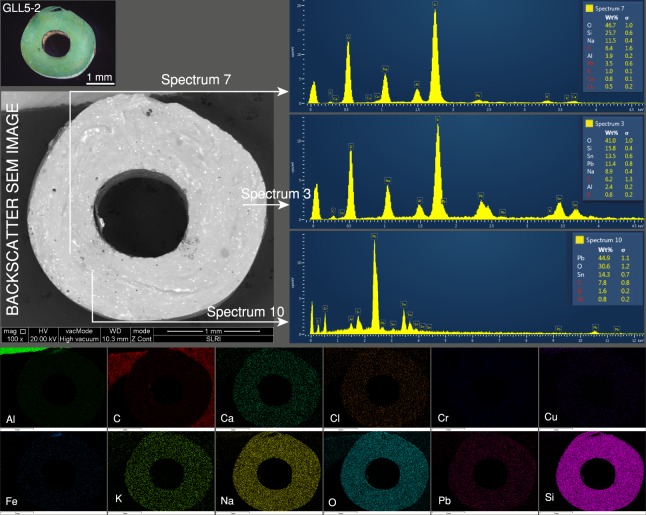
The green sample, GLL5-2, exhibited inhomogeneous textures. Spectrum 7 represents the matrix soda-alumina glass composition. Spectra 3 and 10 were obtained from the inclusions and gave high concentrations of Pb and Sn. The elemental mapping showed higher amounts of Pb.

The green sample, GLL5-2 (Fig. 7), exhibited inhomogeneous textures and flow lines and the bright scattered inclusions were distributed throughout the matrix by the pattern conforming to the flow lines in the glass. Spectrum 7 in the figure was obtained from the clean area and represents the matrix soda-alumina glass composition (O, Si, Na, and Al). Spectra 3 and 10 were obtained for two bright tiny inclusions and show high concentrations of Pb and Sn, suggesting the presence of lead stannate [Pb(Sn,Si)O_3_ or PbSn_1-x_Si_x_O_3_]. The elemental mapping clearly showed that large amounts of Pb were present, while Cu and Fe were found in low concentrations. In this sample, Cu played a role in the primary blue colour of the glass matrix and yellow lead-stannate pigments were added to obtain the secondary green colour (blue + yellow = green).

**Figure 8:**
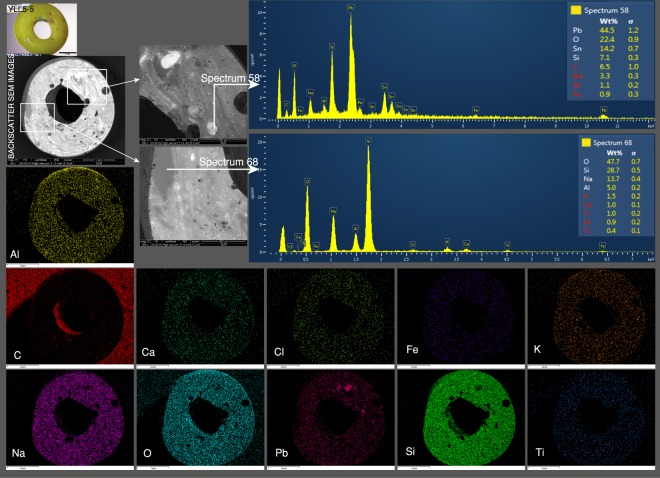
The yellow sample, YLL5-5, shows an abundance of inclusions and flow lines. Spectrum 68 represents the soda-alumina glass matrix. Spectrum 58 is for an inclusion and shows Pb and Sn in the same ratio as that for the spectrum 10 sample (GLL5-2).

The yellow sample, YLL5-5 (Fig. 8), exhibited inhomogeneous texture with abundant inclusions and flow lines. The bright scattered flow lines reflect that the compound does not mix very well with the glass matrix and they contain a large amount of heavier elements. Spectrum 68 in the figure was obtained from the dark grey area and indicated the presence of the soda-alumina glass matrix (O, Si, Na, and Al). Spectrum 58 represents a very bright and large inclusion and shows that the inclusion is very rich in Pb and Sn of the lead-stannate yellow pigments in the same ratio as that of spectrum 10 of the green sample (GLL5-2). This suggests that the same recipe of the pigments was used for the yellow and green glass. It was observed that the Cu in the yellow glass was not suitable and was absent from the elemental mapping list, except for the abundance of the yellow pigment.

**Figure 9:**
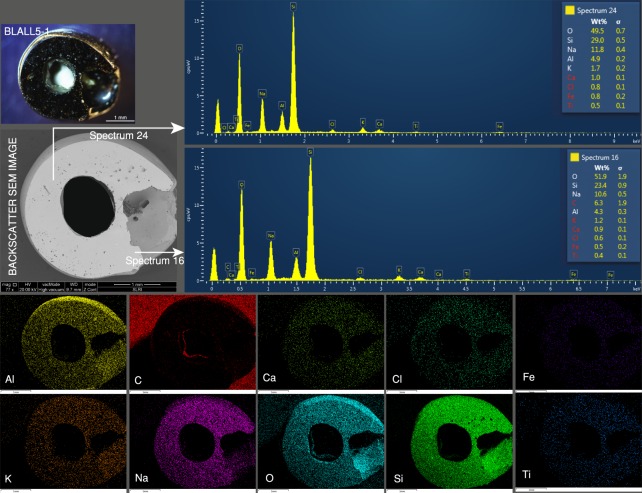
The black sample, BLALL5-1, shows a smooth and clean surface, spectra 16 and 24 reflected the soda-alumina glass matrix. The sample is influenced by Fe observed in the greater abundance in the mapping than for the other coloured glasses.

The black sample, BLALL5-1 (Fig. 9), shows a smooth and clean polished surface under the BSE image, even though there are some bubbles. Spectra 16 and 24 in the figure reflected the matrix of soda-alumina glass (O, Si, Na, and Al), and some other non-significant elements, e.g., K and Fe, were also present. The black colour of the glass is believed to be influenced by the abundance of Fe possibly together with Ti, as observed in the higher abundance of these elements in the elemental mapping images compared to the elemental mapping images of the other coloured glasses.

#### X-ray photo (micro-CT) images

**Figure 10:**
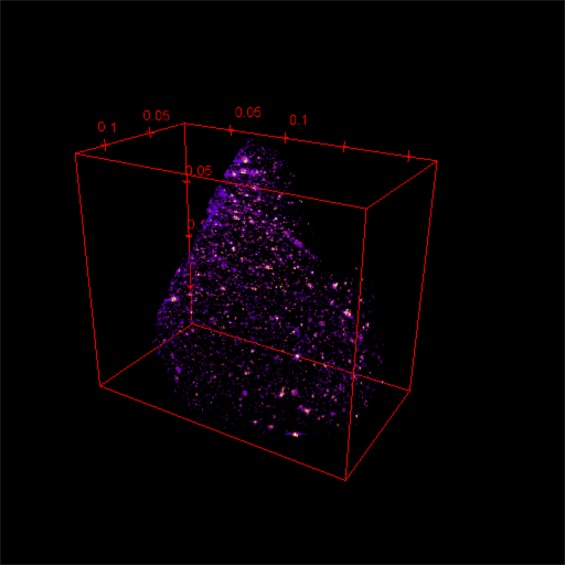
3D Micro-CT images of the glass sample YLL5-4 revealed a random distribution of the bright pigment spots of lead stannate in the glass matrix because they are opaque to the X-ray energy, indicating that the pigments contained very high atomic mass elements, e.g., Pb and Sn, in agreement with the EDS results. (The series of scans and three movie clip files are in the Supplementary Information).

The images obtained from micro-CT (Fig. 10) proved that X-ray opaque materials were dispersed randomly through the matrix of the yellow glass material, YLL5-4. The white spot in Fig. 10 corresponds to the location of high X-ray absorption and indicates the location of high atomic number elements. We assume that the bright spots in micro-CT were Pb because Pb was the heaviest element detected by EDS. Micro-CT revealed that Pb was randomly distributed throughout the sample and was not present only at the surface.

#### Raman spectroscopy

**Figure 11:**
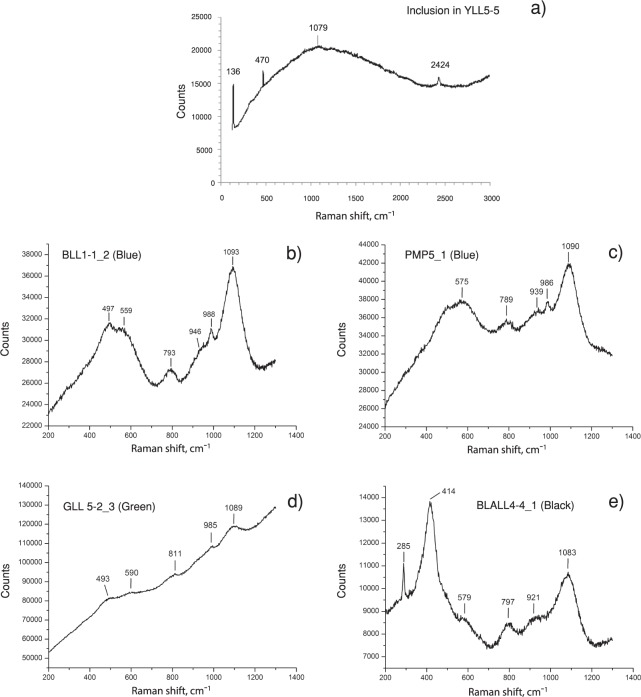
Raman spectra (**a**) for an inclusion in a yellow glass sample, YLL5-5, had a peak of 136 cm^−1^ indicating the presence of lead tin yellow type II (PbSn_1-x_Si_x_O_3_). The band at 470 cm^−1^ is a cosmic spike but other peaks were due to the glass; (**b**–**d**) show the peaks assigned to the glass matrix as the Raman beam hit the bulk composition of the samples to obtain the peaks mainly responsible for Si-O bonding (see text). The peak at 414 cm^−1^ of the black sample was attributed to the Fe-S chromophore and that at 285 cm^−1^ was attributed to the metastable iron minerals.

The Raman shifts were analysed for the bulk samples for some of the samples investigated in this project, as has been reported elsewhere. In this study, the laser beam hit an inclusion of a yellow sample, and the strongest peak at 136 cm^−1^ is typical for lead tin yellow type II (PbSn_1-x_Si_x_O_3_)^20^ (Fig. 11). For the blue, green, and black samples, the Raman shifts of the bulk composition of the glass matrix were also present in the same figure. According to Dussauze *et al*.^21^, the bands at about 450-550 cm^−1^ were assigned to the mixed stretching-bending vibration of Si-O-Si bridges, the band at about 800 cm^−1^ to the bending mode of Si-O-Si bridges, and the higher frequency bands from 940 to 1100 cm^−1^ to the stretching vibration of Si-O- non-bridging bonds in silicate tetrahedral units. The black samples showed a peak at ∼414 cm^−1^, possibly from the chromophore of Fe-S^16,22^ and the peak at 285 cm^−1^ due to metastable iron minerals^23^. Sulphur was below the detection limit of the EDS instrument used in this study.

The SEM-EDS images revealed that lead-stannate pigments were not added to the blue and black glasses, but zircon and cassiterite inclusions were found in very slight amounts. These inclusions were most likely formed by the melting process or alternatively were unintentionally added during the preparation process, for example, the minerals can be an impurity in the silica-sand ingredient. Nevertheless, it is possible that cassiterite was intentionally added as an opacifier^8^. In a previous study^3^, it was shown that the yellow samples were closely matched to Garumele yellow beads^24^, in terms of a high PbO content. The blue glass had a Cu-colouring element, while the black glass had Fe as the colouring element. The XANES experiments clearly proved that the blue colouring element was in the form of Cu^2+^ (evidence for Group II: Deep blue samples, BLL1-1, BLL1-3, and BLL1-4, and PMP-7) and was possibly linked to the oxygen in the form of CuO that gave the glass its blue colour. The black and yellow samples contained only Cu^+^ (for example, represented by Group I: Black colour, BLALL5-1, and Group IV: the yellow samples, YLL5-3, YLL5-4, and YLL5-5) and may be found in the glass as Cu_2_O. XANES measurements proved that the colouring ion in all of the black samples was Fe^2+^ and that it probably bonded with the oxygen in the form of FeO and gave rise to the black colour in the sample. Elemental mapping revealed that the Cu and Fe were evenly distributed throughout the glass body, suggesting that these elements bonded with the oxygen in the glass-forming network, probably acted as a non-bridging oxygen and did not act as the pigment^25^. The Mn content in the study samples was too low to be detected by EDS but the XANES results showed that the samples tended to serve as Mn^3+^ in the glasses. However, in this stage, the Mn does not contribute to the colour of the glass. The green glass has hybrid colours between blue and yellow due to Cu, which plays a major role for the primary blue, while the lead stannate played a major role of the yellow, and then these two sources blended to create a secondary green colour. This suggested that glass makers could possibly obtain glass products in different desired shades of blue-green, green, yellowish-green, greenish-yellow by varying the amount of the lead stannate pigment added to the Cu^2+^−bearing melt.

The colour of the yellow glass can only be provided by lead stannate, and larger amounts of the pigments were found compared to the green samples. The pigments did not mix well with the matrix glass, as seen in the dispersed inclusions and concentration on the flow lines in both green and yellow glasses. However, they can partially dissolve in the glass as observed in the distribution of Pb in the mapping image of the yellow and green samples (Fig. 7, GLL5-2 and Fig. 8, YLL5-5). The high Pb content led to the highest specific gravity of the yellow samples^3^. The lead stannate in this study was the lead-tin yellow type II [Pb(Sn,Si)O_3_ or PbSn_1-x_Si_x_O_3_] that was deliberately added to the glass melt as the colourant and opacifier^8,26^.

The contemporary samples in the reign of Dvaravati (6^th^–11^th^ centuries) examined in this study were obtained from the highland (Pang Mapha, Mae Hong Son) and the lowland (Sa Kaeo) areas of Thailand that shared the same technology, particularly for the blue and blue green glasses (PMP vs. BLL). The yellow samples from Sa Kaeo can be compared with the samples from China studied by Li *et al*.^8^ in terms of the use of lead-tin yellow type II and the cassiterite found in the greenish-blue sample from Pang Mapha. This suggests that the trade in ancient glass beads could connect to China at the time period mentioned. The tin-based opacifiers were reported to be first used in glasses in Europe in 200–100 BC^27,28^. Later, in the 4^th^–18^th^ centuries, they were widely used again in glass production, and the glasses were traded all over the world^29^. The glasses examined in this study could possibly be imported or technologically transferred to domestic manufacturers at that time during the period of trade on the Silk Road that connected the East and the West.

## Conclusions

The Dvaravati glasses can be classified based on the colouring elements/compounds as Cu^2+^ for blue glasses, Cu^2+^ and lead stannate for green and blue-green glasses, lead stannate for yellow, and Fe^2+^ for black glasses. The XANES study revealed that the black glasses contained Cu^+^, the blue glasses contained mixed proportions of the Cu^+^ and Cu^2+^, the greenish-blue glasses contained mixed proportions of Cu^+^ and Cu^2+^, but Cu^+^ is more abundant, and the yellow and green glasses contained Cu^+^. SEM-EDS, X-ray photo (micro-CT), and Raman microscopy were useful for disclosing the pigments such as lead stannate or lead tin yellow type II (PbSn_1-x_Si_x_O_3_) and other inclusions, i.e., cassiterite (SnO_2_) and zircon (ZrSiO_4_) in the glasses. The pigment domains in the yellow and green glasses gave rise to the inhomogeneous matrix and the yellow pigments can be clearly observed. The studied samples identified a link between Chinese trade in ancient times, probably via the Silk Road that connected the East and the West.

## Methods

The 45 samples in this study were selected from 203 specimens. They ranged from 2 to 5 mm across and were various colours, i.e., blue, green, yellow and black, and their appearance is shown in the figures in this paper. All of the samples were collected from the archaeological sites of two provinces, from the log-coffin caves at Pang Mapha, Mae Hong Son Province, dated back to ~2100–1200 BP, and the contemporary age of the Dvaravati tomb at Sa Kaeo Province, Thailand^30,31^. The samples are “m-Na-Al” glass (m = mineral, Na = soda, Al = alumina)^32^. The locations and details of the samples were reported in Saminpanya *et al*.^3^, and the prefixes of sample identification were as follows: PMP = Pang Mapha, Mae Hong Son Province (colours ranging from blue, greenish blue, bluish green to yellowish green); GLL = green from Nong Pak Waen at Ta Phraya, Sa Kaeo Province; YLL = yellow from Nong Pak Waen at Ta Phraya, Sa Kaeo Province; BLALL5-1 to 4 = black from Nong Pak Waen at Ta Phraya, Sa Kaeo Province; BLALL4-2 to 4 = black from La Lu at Ta Phraya, Sa Kaeo Province; BLL = blue from Khao Sam Sip at Khao Chakan in the Sa Kaeo Province.

SEM-EDS scanning electron microscopy equipped with energy dispersive spectrometry, Oxford Instruments, X-Max, was used in the window of the Silicon Drift Detector with a size of 50 mm^2^ and resolution of 127 eV. The yearly routine calibrations were performed by using the pure metal elements. This technique was used to observe the texture and the inclusion of the glass via backscattered electron (BSE) images to qualitatively analyse the chemical compositions and to perform the elemental mapping of the samples. Prior to the analysis, the surfaces of the samples were polished to make a flat window in order to obtain a pristine matrix and the exposure of the inclusion. The experimental parameters for the instrument (FEI QUANTA 450) used to obtain the BSE images were as follows: accelerating voltage of 20 keV, pressure of 1.560–2.000 Pa, and working distances between 9.7 and 21.9 mm, depending on the magnification. The EDS instrument was connected to the Aztec 3.3 computer software.

Elemental analysis, oxidation state and the tomography of the samples were obtained using synchrotron-based X-ray techniques at the Synchrotron Light Research Institute (SLRI), Thailand. The SLRI storage ring is operated at the electron energy of 1.2 GeV with the electron beam current of 80–150 mA. The synchrotron radiation from a 2.2 Tesla multipole wiggler was monochromatized by using a commercial double-crystal X-ray monochromator equipped with Si(111) crystals at beamline 1.1 W (BL1.1 W). Furthermore, polychromatic X-ray beams in the energy range from 4 to 20 keV were used at beamline 1.2 W (BL1.2 W). The measurements were conducted using three synchrotron techniques using two beamlines at the SLRI.

1. XRF was measured at BL1.1 W to obtain the whole elemental fluorescence spectra emitted from core-level electron excitation, using the photon energies of 10 keV and 14 keV. A highly sensitive Canberra 19-element Ge solid-state detector was used to record the XRF spectra with an accumulation time of 2 minutes. The spectral weights of Cu and Fe were calculated using the PyMCA program in order to semi-quantitatively assess the elemental contents^33^.
2. Some samples were then selected for analysis by X-ray absorption near edge structure (XANES) technique to evaluate the valences of the transition metals with a focus on Cu, Fe, and Mn. The XANES spectra were recorded in the fluorescence yield mode using the same detector used in the XRF experiments with a 0.2 eV energy step near the absorption edge. The foil standards were used to calibrate X-ray energies for each element by setting the absorption edges of Cu, Fe and Mn to 8979 eV, 7112 eV, and 6539 eV, respectively. CuO, Cu_2_O, CuSO_4_, FeO, Fe_2_O_3_, goethite (α-Fe^3+^O(OH)), MnO, Mn_3_O_4_, Mn_2_O_3_, and MnO_2_ were measured in the transmission mode using ionization chambers. To determine the unknown oxidation state of the sample, the positions of the absorption edges of the samples were compared to those of the standard. At least two spectra from each sample were merged to ensure precision and to obtain high signal-to-noise levels.
3. X-ray tomographic microscopy (XTM) at BL1.2 W was used to obtain X-ray photo images of a fraction of YLL5-4 sample providing 3D images with high contrast inclusions inside the sample. We chose this sample because it contains a large amount of inclusions. An experimental setup was used in the mode of SRXTM or Micro computed tomography (Micro-CT) with an exposure time of 100 ms, scan angle of 0–180.00°, angular increments of 0.100° and pixel size of 1.44 *μm*. The calibration bar was based on the dynamic range of 8-bit images (0–255 as a function of X-ray absorption of the sample). The sample compositions were segmented for minimum X-ray absorption.

A Renishaw inVia Raman microscope was used to obtain Raman spectra and confirm the results obtained by other techniques. The conditions of the equipment include the use of a green laser line at 532 nm, grating at 1800 nm; exposure time of 10 sec/run in the extended mode, accumulation of 10 runs, Raman shift in the 120–3000 (cm^−1^) range, laser power of 16 mW at the sample, and a magnification for the objective lens of 50×.

## Supplementary information

Supplementary Information - Shedding New Light on Ancient Glass Beads by Synchrotron, SEM-EDS and Raman Spectroscopy Techniques
