# Supplementary material for: Shedding New Light on Ancient Glass Beads by Synchrotron, SEM-EDS, and Raman Spectroscopy Techniques
Source: Sci Rep. 2019 Nov 5;9:16069. doi: 10.1038/s41598-019-52322-2 (PMC6831571; doi:10.1038/s41598-019-52322-2)
Supplement: Supplementary file 1 — Supplementary Information - Shedding New Light on Ancient Glass Beads by Synchrotron, SEM-EDS and Raman Spectroscopy Techniques [file 41598_2019_52322_MOESM1_ESM.pdf]

Supplementary Information

Shedding New Light on Ancient Glass Beads by Synchrotron, SEM-EDS, and Raman Spectroscopy Techniques

Seriwat Saminpanya*, Chatree Saiyasombat, Nirawat Thammajak, Chanakarn Samrong, Sirilak Footrakul, Nichanan Potisuppaiboon, Ekkasit Sirisurawong, Thumrongsak Witchanantakul, Catleya Rojviriya

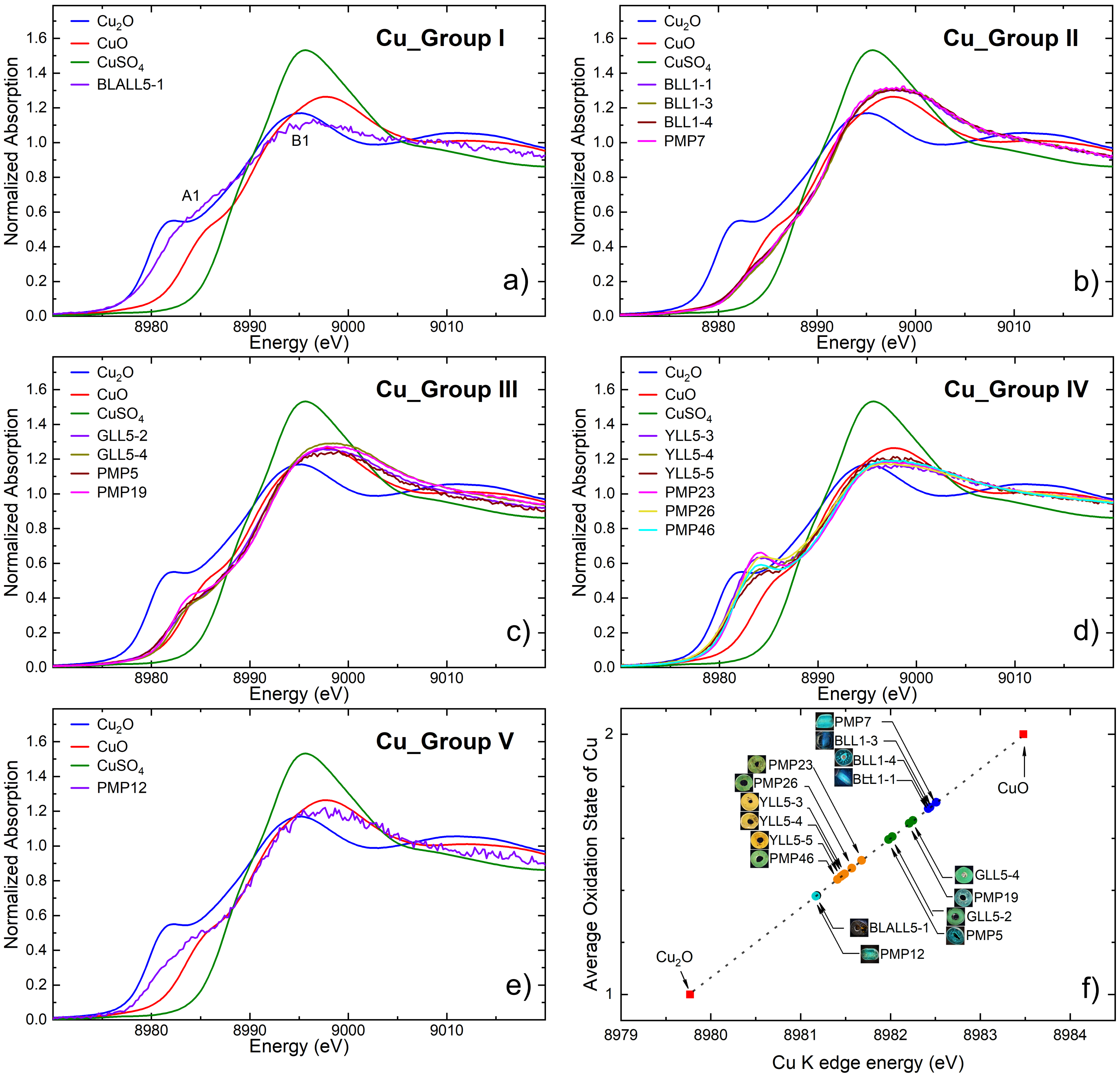

Supplementary Figure 1. (a) - (e) Cu K-edge XANES spectra for each group; (f) a summary of the average values for the Cu oxidation states plotted against the K-edge energy derived from the experiments.

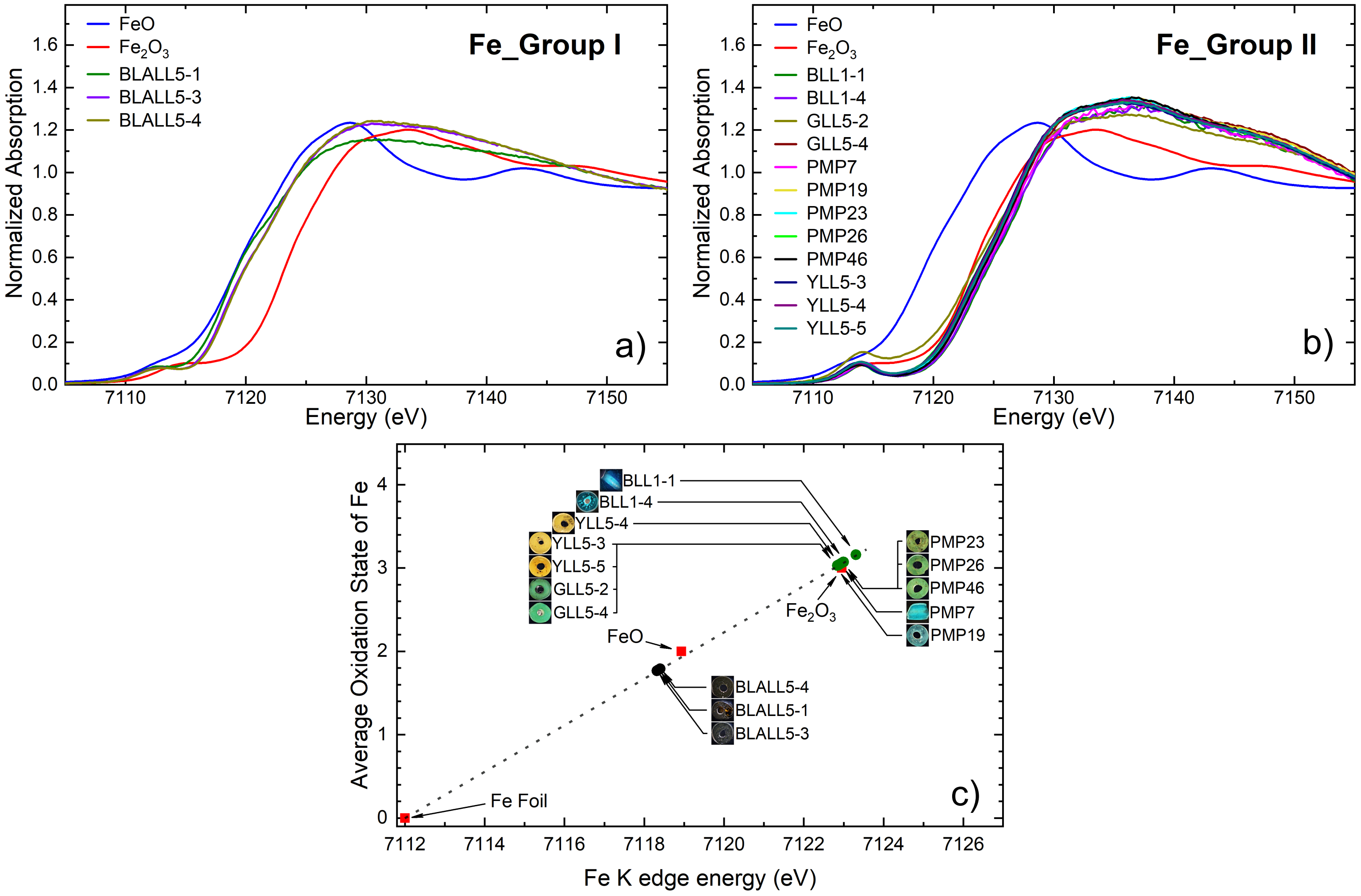

Supplementary Figure 2. (a) and (b)) Fe K-edge XANES spectra for each group; (c) a summary of the average values for the Fe oxidation states plotted against the K-edge energy derived from the experiments.

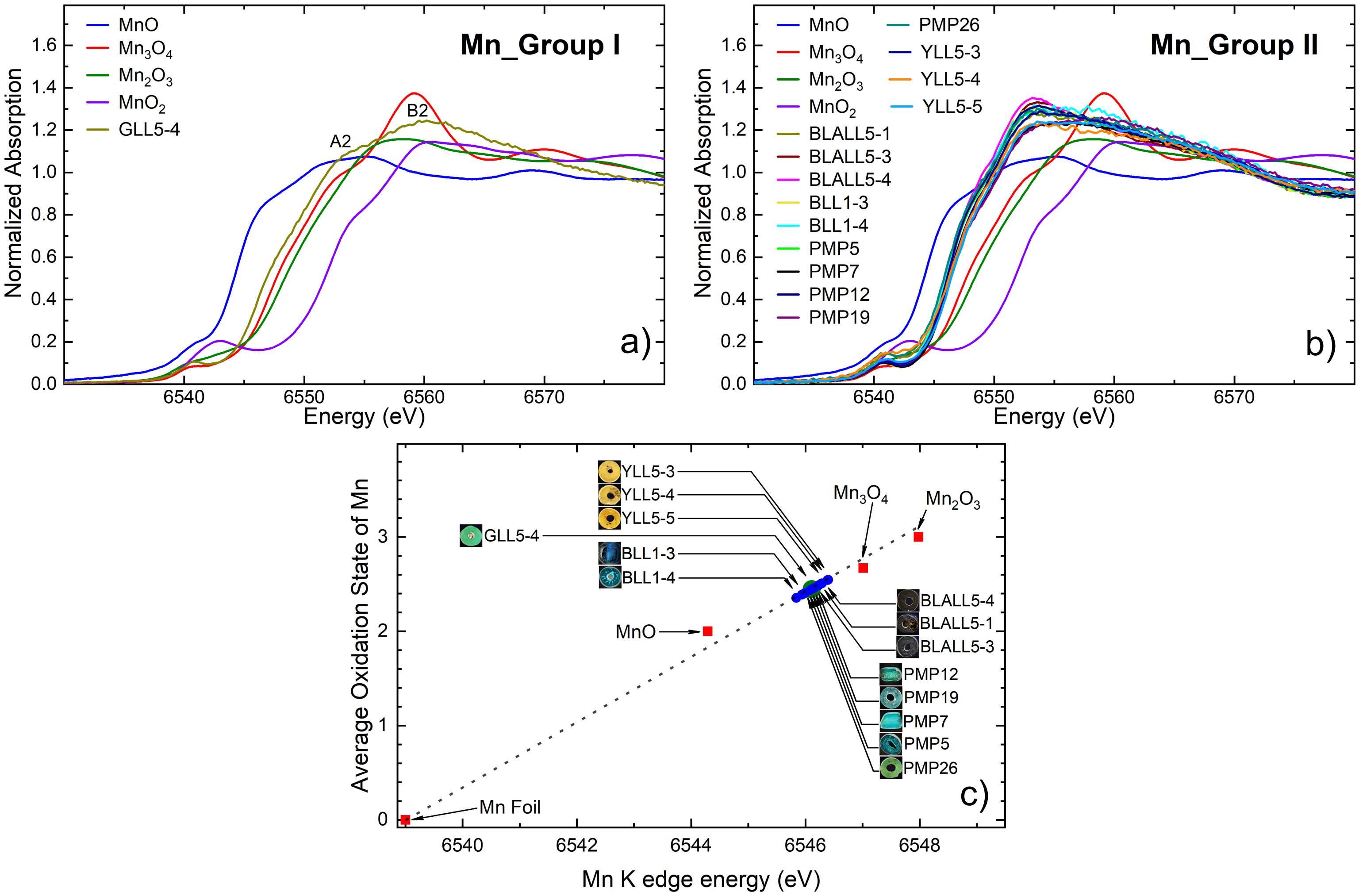

Supplementary Figure 3. (a) and (b)) Mn K-edge XANES spectra for each group; (c) a summary of the average values for the Mn oxidation states plotted against the K-edge energy derived from the experiments.

Suppementary Tables

Table AA The table shows the absorption edge and average oxidation states of Cu for studied samples.

Name	E0	Average oxidation state
Cu foil	8979.00	0.00
Cu2O	8979.77	1.00
CuO	8983.48	2.00
BLALL5-1	8981.18	1.38
Cu BLL1-1	8982.42	1.71
Cu BLL1-3	8982.44	1.72
Cu BLL1-4	8982.43	1.72
Cu PMP7	8982.51	1.74
Cu GLL5 2	8982.02	1.61
Cu GLL5 4	8982.25	1.67
Cu PMP5	8981.98	1.60
Cu PMP19	8982.21	1.66
Cu YLL5 3	8981.49	1.46
Cu YLL5 4	8981.47	1.46
Cu YLL5 5	8981.45	1.45
Cu PMP23	8981.68	1.52
Cu PMP26	8981.57	1.49
Cu PMP46	8981.41	1.44
Cu PMP12	8981.17	1.38

Table AB The table shows the absorption edge and average oxidation states of Fe for studied samples.

Name	E0	Average oxidation state
Fe Foil	7112.00	0.00
FeO	7118.93	2.00
Fe2O3	7122.95	3.00
BLALL5-1	7118.34	1.77
BLALL5-3	7118.32	1.77
BLALL5-4	7118.40	1.79
BLL1-1	7123.31	3.16
BLL1-4	7123.00	3.07
GLL5-2	7122.84	3.03
GLL5-4	7122.86	3.03
PMP7	7122.96	3.06
PMP19	7122.84	3.03
PMP23	7122.99	3.07
PMP26	7122.99	3.07
PMP46	7122.99	3.07
YLL5-3	7122.86	3.03
YLL5-4	7122.91	3.05
YLL5-5	7122.87	3.04

Table AC The table shows the absorption edge and average oxidation states of Mn for studied samples

Name	E0	Average oxidation state
Mn Foil	6539.00	0.00
MnO	6544.29	2.00
Mn3O4	6547.01	2.67
Mn2O3	6547.98	3.00
Mn_GLL5-4	6546.11	2.45
Mn_BLALL5-1	6546.23	2.49
Mn_BLALL5-3	6546.15	2.46
Mn_BLALL5-4	6546.28	2.51
Mn_YLL5-3	6546.40	2.55
Mn_YLL5-4	6546.39	2.54
Mn_YLL5-5	6546.29	2.51
Mn_PMP5	6546.08	2.44
Mn_PMP7	6546.11	2.45
Mn_PMP12	6546.12	2.45
Mn_PMP19	6546.12	2.45
Mn_PMP26	6545.99	2.41
Mn_BLL1-3	6545.84	2.35
Mn_BLL1-4	6545.94	2.39

Supplementary Videos

3 Video clips of the 3D Micro-CT scan.

https://drive.google.com/file/d/1vEaIESxuaub4TY8N4spvGG26OE_rQLk8/view?usp=sharing

https://drive.google.com/file/d/1yNQiej5iHSKDp0w5QWWgQmNleWpGIenV/view?usp=sharing

https://drive.google.com/file/d/14303gRACP1rUTZrmzR7ze_g_5DLMTpbU/view?usp=sharing

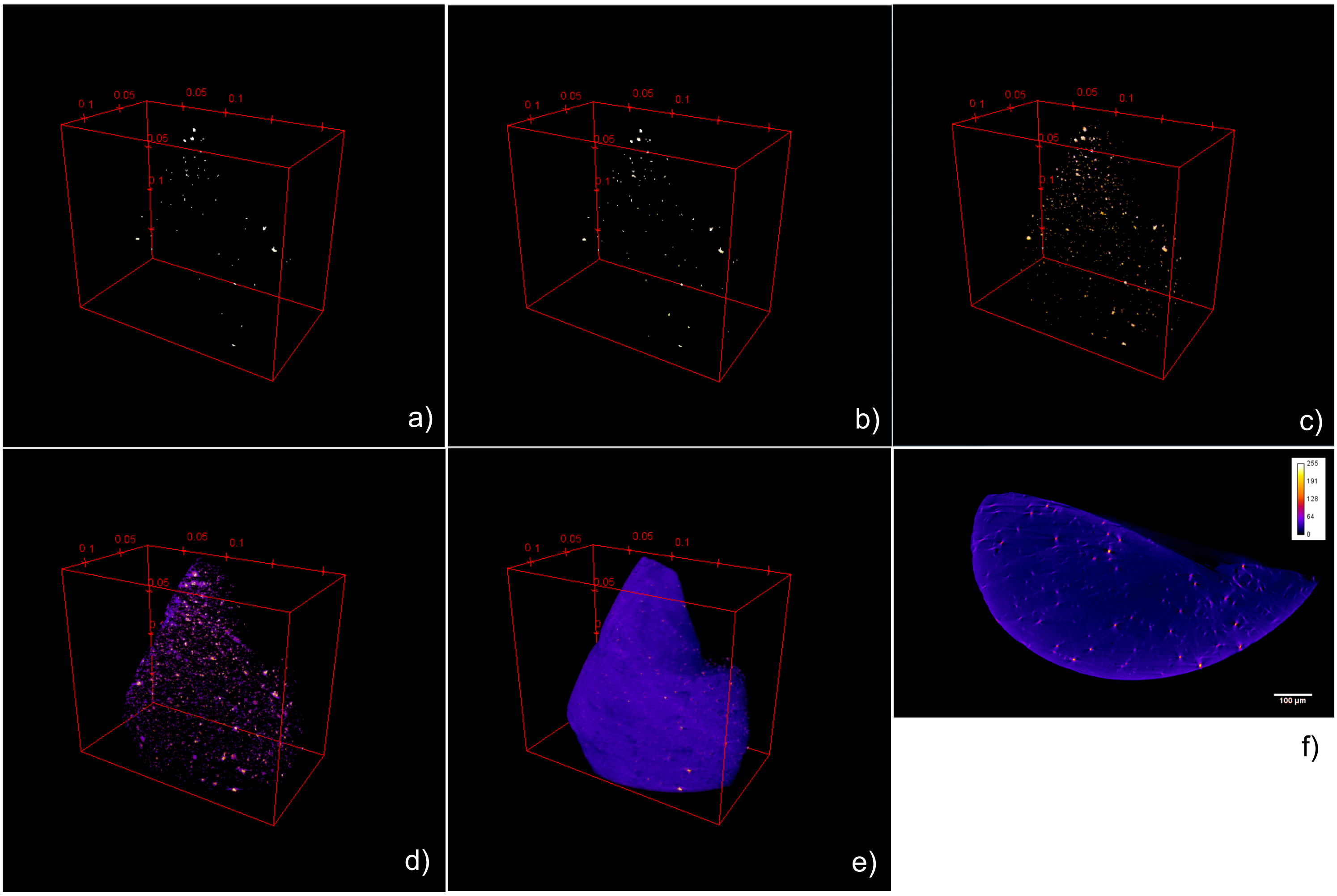
a)
b)
c)
d)
e)
f)
0.1
0.05
255
191
128
64
0
100 µm
